# A Multi-Methodological Protocol to Characterize PDO Olive Oils

**DOI:** 10.3390/metabo8030043

**Published:** 2018-07-28

**Authors:** Simone Circi, Cinzia Ingallina, Silvia Vista, Donatella Capitani, Andrea Di Vecchia, Genesio Leonardi, Giovanni D’Achille, Luigi Centauri, Federica Camin, Luisa Mannina

**Affiliations:** 1 Dipartimento di Chimica e Tecnologie del Farmaco, Sapienza Università di Roma, P.le Aldo Moro 5, 00185 Rome, Italy; simone.circi@uniroma1.it (S.C.); cinzia.ingallina@uniroma1.it (C.I.); vistasilvia@gmail.com (S.V.); 2 Istituto di Metodologie Chimiche, CNR, Area della Ricerca di Roma 1, Laboratorio di Risonanza Magnetica “Annalaura Segre”, Via Salaria km 29,300, 00015 Monterotondo, Italy; donatella.capitani@cnr.it; 3 Istituto di Biometeorologia, CNR, Via dei Taurini 19, 00185 Rome, Italy; a.divecchia@ibimet.cnr.it; 4 Associazione Produttori Olivicoli Latina, via Don Minzoni 1, 04100 Latina, Italy; capol.latina@gmail.com (G.L.); giovanni1111@vodafone.it (G.D.); 5 Centro Assaggiatori Produzioni Olivicole Latina, via Don Minzoni 1, 04100 Latina, Italy; lcentauri@regione.lazio.it; 6 Department of Food Quality and Nutrition, Research and Innovation Centre, Fondazione Edmund Mach (FEM), via E. Mach 1, 38010 San Michele all’Adige, Italy; federica.camin@fmach.it

**Keywords:** olive oil, panel test, IRMS, NMR

## Abstract

An analytical approach including Panel Test, Isotope Ratio Mass Spectrometry (IRMS) and Nuclear Magnetic Resonance (NMR) spectroscopy was proposed to characterize Italian “*Colline Pontine*” PDO olive oils (40 samples) of two consecutive crop years. Our approach has evidenced the high quality of these olive oils. Only 6 of 40 olive oils samples were defined as “*defective*” by the official Panel Test due to the detection of negative sensory attributes. The low variability of isotopic data monitored by IRMS confirmed that the olive oil samples all came from a limited geographical area. NMR spectra did not evidence any chemical composition anomaly in the investigated samples. In order to assess the influence of harvesting year over the olive oil chemical composition, the NMR analysis was extended to other 22 olive oil samples of a third harvesting year. NMR data were submitted to two different statistical methods, namely, analysis of variance (ANOVA) and principal component analysis (PCA) allowing olive oils of three consecutive harvesting years to be grouped.

## 1. Introduction

Foodstuff authentication and geographical origin certification [1,2] is an actual issue and an important challenge faced by different analytical methodologies. Regarding olive oils, a mandatory labeling reporting the geographical origin is required by the EU Regulation 182/11 [3,4].

The olive growing area of the district of Latina, recognized as PDO “*Colline Pontine*” in 2009, extends behind the *agro Pontino*, developing parallel to the sea, through the mountain systems of Lepini, Ausoni and Aurunci Mounts. The “*Colline Pontine*” PDO area is one of the wider Italian olive growing areas characterized by the constant presence of the “*Itrana*” cultivar, which is used for olive oil and table olives production (Gaeta’s olive).

In the last years, the emergence of producers who have brought innovation to the traditional production process has allowed olive oil of the PDO “*Colline Pontine*” area to achieve excellence aims highlighted by both prestigious awards obtained and by consumer responses. This aspect is at the origin of the project “*Quality olive growing and territory*” in the PDO “*Colline Pontine*” area, developed under the program “*Integrated Skills for Sustainability and Innovation of Food Made in Italy*” promoted by the Italian Ministry of Finance.

Today both chemical and sensorial analyses represent the official methods to define the commercial value of olive oils, as established by EEC Regulation 2568/91 [5].

An important analysis is the Panel test that develops a global picture of olive oil sensory properties. In this way, this analysis, not yet replaceable with other analyses, gives add values to peculiar extra virgin olive oils (EVOOs) by describing their special sensorial characteristics. On the other hand, as discussed in literature [6], in the case of common commercial EVOOs this analysis can give discordant results, turning out to be dependent from panel laboratory.

In recent years many studies have been performed to characterize and classify according to the geographical origin olive oils using different “unconventional” techniques, such as Isotope Ratio Mass Spectrometry (IRMS) [7,8,9] and Nuclear Magnetic Resonance (NMR) [10,11,12,13,14,15,16,17,18] or their combination [19].

The H, O and C isotopic composition of plant materials as olive oils depends on many factors, including geographical conditions (altitude, latitude, distance from the sea, etc.) and climatic conditions (relative humidity, temperature, amount of precipitation) [8,9,20,21].

As reported in literature [22], ^1^H NMR spectroscopy enables the attainment of a good chemical characterization of an olive oil, giving information on major (triglycerides) and minor (aldehydes, terpenes, sterols) [23,24] compounds in a single experiment, with an experimental error which is exactly the same and extremely low for all compounds analyzed.

In this paper, an analytical protocol is reported with the aim of promoting the diffusion of high quality olive growing in the “*Colline Pontine*” PDO area and to create a connection between research and industry. This protocol involves Panel Test along with IRMS and NMR spectroscopy.

## 2. Results and Discussion

In order to obtain a comprehensive characterization of olive oils from “*Colline Pontine*” PDO area 40 olive oils samples of two harvest years were analyzed by means of a multidisciplinary protocol including Panel Test, IRMS, and ^1^H NMR spectroscopy, see Table S1. The effect of the harvesting year was investigated in more details considering other 22 olive oils of a third crop year, see Table S1. These olive oils were analyzed and included in the statistical analysis together with the previous 40 samples.

### 2.1. Sensory Analysis

40 olive oil samples coming from different geographical areas of the district of Latina (Lazio) were assayed according to the official method reported in the EC Regulation 2568/91 [5] and EC Regulation 640/08 [25].

The sensorial analysis recognized 34 of 40 samples as extra virgin olive oils, see Table 1. On the other hand, 6 of 40 samples were defined as “*defective*” due to the detection of negative sensory attributes, namely fusty/muddy sediment (samples 8, 12, 25 and 37), musty/humid/earthy (sample 9), winey/vinegary/acid/sour (sample 33) and rancid (sample 37). Therefore, these 6 samples were classified as virgin olive oils. It is important to note that “tomato” is neither a positive attribute nor a negative attribute and therefore it does not affect the score value. This term is specific to evidence and appreciate a “nose-sensed” feature of olive oils.

### 2.2. Isotope Ratio Mass Spectrometry

The results of stable isotope ratios of H, O and C carried out on 40 olive oil samples are reported in Table 2. As expected for samples produced in a narrow area, we did not find a large variability of the isotopic data, with δ^2^H ranging from −152 to −135‰, δ^18^O from 22.0 to 26.3‰ and δ^13^C from −31.9 to −28.1‰. These values may be considered characteristic of the PDO “*Colline Pontine*” olive oils.

### 2.3. Nuclear Magnetic Resonance Spectroscopy

**Figure 1:**
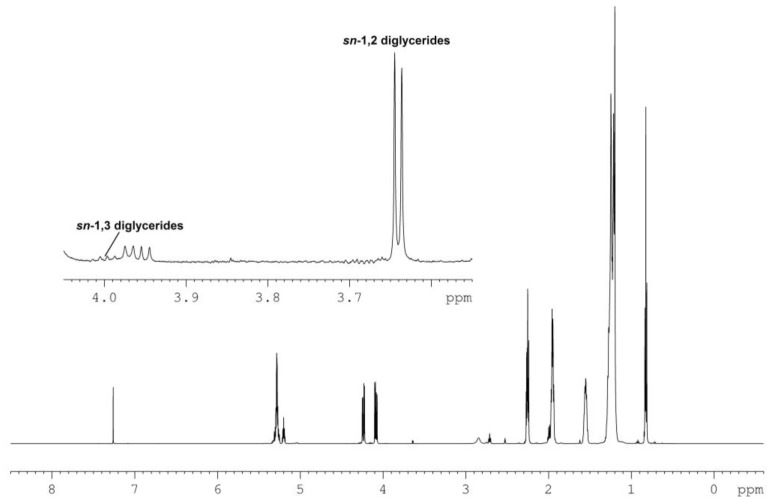
600.13 MHz ^1^H NMR spectrum of an olive oil. An expansion of the 3.5–4.0 ppm spectral region including *sn*-1,2 diglycerides and *sn*-1,3 diglycerides signals, is shown.

Olive oils (62 samples) were submitted to the NMR analysis. The ^1^H NMR spectrum of an olive oil is shown in Figure 1. In the 3.5–4.0 ppm spectral region, the signal due to *sn*-1,2 diglycerides and *sn*-1,3 diglycerides are observable, giving an index of the olive oil freshness [12].

Freshly made olive oil from healthy olive fruits contains almost only *sn*-1,2 diglycerides whose content decreases during the storage, whereas the *sn*-1,3 diglycerides content increases [26] due to the gradual isomerization of *sn*-1,2 diglycerides to *sn*-1,3 ones. Different factors, such as the extraction process, shelf-life, storage conditions, presence of macromolecules, metals are responsible for the *sn*-1,2/1,3 diglycerides ratio [27,28]. Therefore, this ratio is strongly related to the freshness of an olive oil [10,29]. In fresh olive oils, this ratio is greater than 4, whereas a ratio < 4 indicates a low-quality olive oil [30].

In our case, all olive oil samples possessed a *sn*-1,2/*sn*-1,3 diglycerides ratio greater than 4, suggesting a good quality product and a suitable storage process, see Table 3.

### 2.4. Statistical Analysis

In order to assess the harvesting year influence over the olive oil chemical composition, a statistical analysis was performed: 13 selected resonances (see Table S2) sensitive to the geographical origin [22] were carefully measured and the normalized intensities were submitted to a suitable statistical analysis.

**Figure 2:**
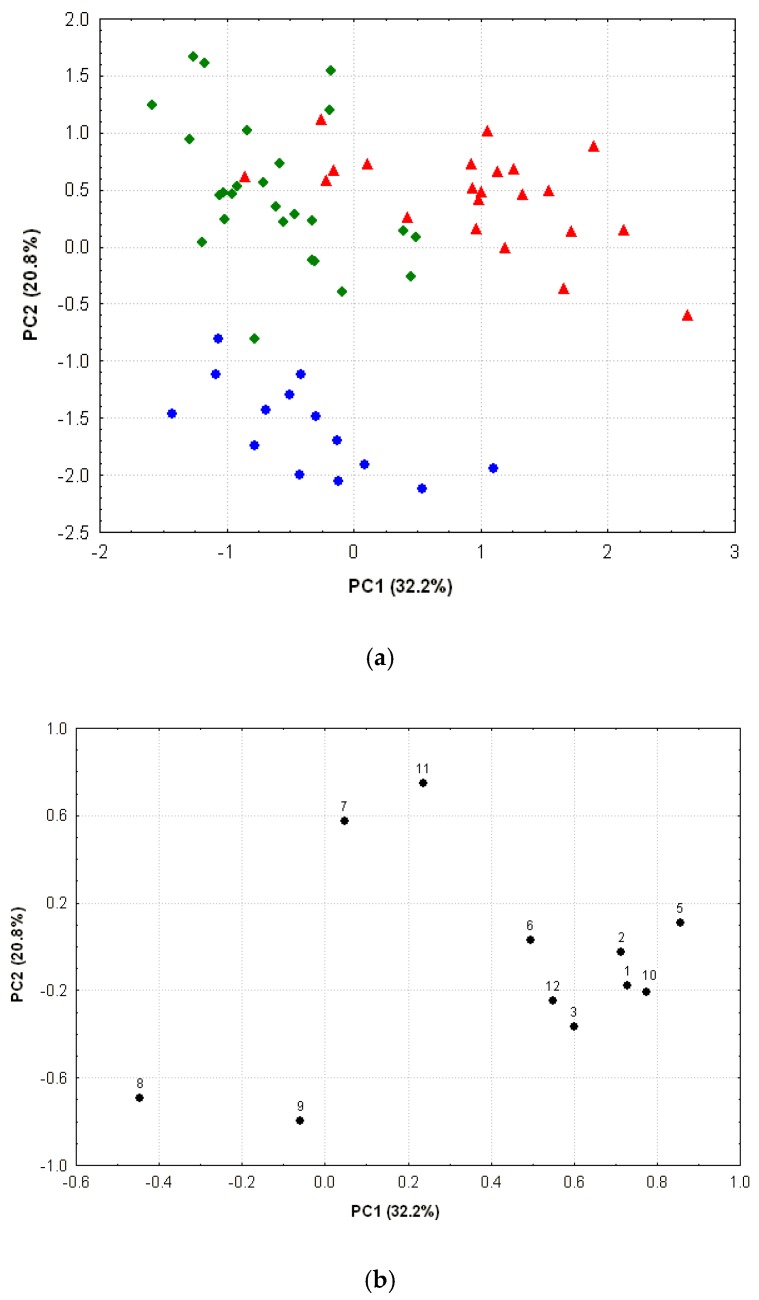
(**a**) Score plot of PCA along the two main components performed on NMR data regarding 62 olive oil samples, produced during three harvesting years labelled as blue circles, green diamonds, red triangles according to the respective year time order. The eleven variables with the highest discrimination power selected by ANOVA were used for the PCA and labelled by the Arabic numbers 1–3 and 5–12 (explained in the text); these are displayed in the (**b**) analogous plot of loadings.

The ANOVA was performed on the intensity of the selected resonances to find the variables with the highest discriminating power. Except for “Terpene 1” (4) and “β-sitosterol” (13), the remaining eleven variables showed to be significant for the characterization of olive oil samples and they were submitted to the PCA analysis. Terpene 1 seems to represent a variable independent from the harvesting effect because its content was found to be, on average, constant in the three different harvest years. The PCA map, labelled according to the three harvesting years, is shown in Figure 2a. The PC1, explaining 32.2% of the total variance, facilitates observation of a good separation between olive oil samples from the first two harvesting years and the third harvesting year. The PC2, explaining 20.8% of the total variance, shows a discreet separation between olive oil samples from the first harvesting year and the other two ones.

The most discriminant parameters correspond to saturated (9) and unsaturated (8) fatty acids, having a high value in samples of the first harvesting year, to linolenic acid (11) and squalene (7), having a high value in samples of the second and the third harvesting years, and to 24-methylene cycloartanol (10), *sn*-1,3 diglycerides (5) and the two aldehydes (1, 2), having a high value in samples of the third harvesting year (see the corresponding loadings shown in Figure 2b).

The variability of all these compounds in the three harvests can be due to different factors such as the different climatic conditions.

## 3. Materials and Methods

**Figure 3:**
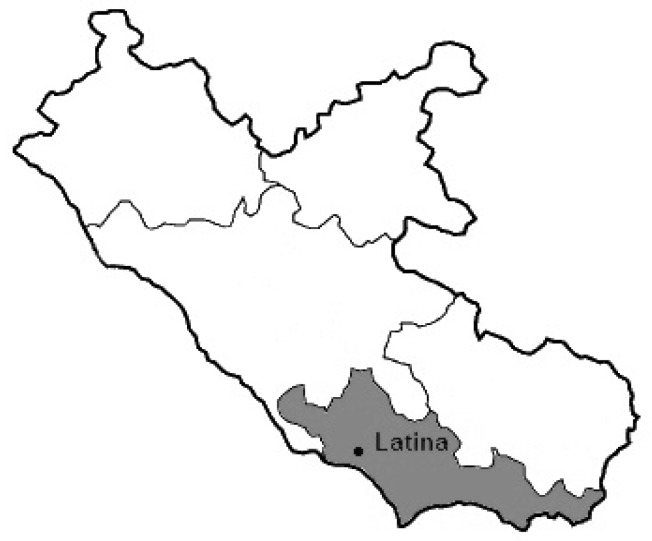
Map of Lazio region (Italy): the area of Latina district is highlighted.

The basic characteristics of 62 olive oil samples produced in different areas of the district of Latina, Lazio (see Figure 3), are reported in Table S1. Olive oils were extracted from olive batches from known place, production years and variety.

The sampling consists of 14 monovarietal olive oils (13 Itrana and 1 Carolea) of a first harvest year, 26 monovarietal and multivarietal olive oils (24 Itrana, 1 Carolea, and 1 Itrana, Frantoio) of a second harvest year and 22 monovarietal olive oils belonging to the cultivar Itrana of a third harvest year.

### 3.1. Sensory Analysis

Sensory analysis on 40 olive oil samples from the first and the second crop years was performed in the tasting room of “Camera di Commercio” of Latina by a fully trained analytical taste panel, made up of a panel head and 9 tasters, and recognized by the International Olive Council (IOC). The evaluation of the samples was carried out according to the official method described in the EC Regulation 2568/91 [5] and the EC Regulation 640/08 [25]. Samples were stored in 0.50 L bottles, having the same color and shape and without any reference to the producer. Each taster smelled and tasted the oils in order to analyze positive and negative attributes. Each assessor rated the intensity of the sensory attributes using a unstructured scale of 10 cm, ranging from low to high intensity. Results were expressed as the median intensity of the sensory perceptions of the tasters.

In the case of olive oils coming from the district of Latina the attribute “tomato” was added to the positive attributes. In fact, “tomato” is a typical sensory attribute that characterized olive oils of the PDO “*Colline Pontine*” area.

### 3.2. Isotope Ratio Mass Spectrometry

The analysis of the ratios ^13^C/^12^C, ^18^O/^16^O and ^2^H/^1^H of bulk olive oils was performed on the same forty samples submitted to the sensory analysis.

The ratio ^13^C/^12^C was measured using an Isotope Ratio Mass Spectrometer (DELTA V, Thermo Scientific, Langenselbold, Germany) following total combustion in an elemental analyzer (EA Flash 1112, Thermo Scientific). The ratios ^2^H/^1^H and ^18^O/^16^O were measured in one run using IRMS (Finnigan DELTA XP, Thermo Scientific) coupled with a Pyrolyser (Finnigan TC/EA, high temperature conversion elemental analyzer, Thermo Scientific), following the methods described elsewhere [7].

The values were expressed as following:

δ‰ = [(R_sample_ − R_standard_)/R_standard_] (1)

where R is the ratio between the heavier isotope and the lighter one.

The values were expressed in δ‰ against international standards Vienna-Pee Dee Belemnite (V-PDB) for δ^13^C, Vienna–Standard Mean Ocean Water (V-SMOW) for δ^2^H and δ^18^O. For the calculation of δ‰, 2 olive oil working inhouse standards were calibrated against international reference materials: fuel oil NBS-22 (IAEA) and sugar IAEA-CH-6 (IAEA) for ^13^C/^12^C, Benzoic Acid IAEA-601 (IAEA) for ^18^O/^16^O, fuel oil NBS-22 (IAEA) for ^2^H/^1^H, Icosanoic Acid Methyl Esters USGS70 and USGS71 for ^13^C/^12^C and ^2^H/^1^H. 

The standard deviation of repeatability (Sr) for oil was 0.1‰ for δ^13^C, 0.4‰ for δ^18^O and 1‰ for δ^2^H.

### 3.3. NMR Measurements

All the NMR spectra were carried out in December to have fresh EVOO with the same shelf-life.

Olive oil samples (20 μL) were dissolved in CDCl_3_ (700 μL) plus DMSO-d_6_ (20 μL), directly in the 5 mm NMR tube, to solubilize all minor components. For NMR measurements we considered both the forty olive oil samples from the first and the second crop years and the twenty-two samples from the third harvesting year.

^1^H NMR experiments were recorded at 300 K on a Bruker AVANCE 600 NMR spectrometer operating at the proton frequency of 600.13 MHz (B_0_ = 14.1 T) and equipped with a Bruker multinuclear Z gradient 5 mm probe head. The ^1^H spectra were acquired using the following conditions: number of scans 1024, π/2 pulse ~ 8 μs, time domain (TD) 64 K data points, relaxation delay plus acquisition time 3.5 s and spectral width 18.5 ppm. ^1^H NMR spectra were obtained by the Fourier transformation of the Free Induction Decay, applying an exponential multiplication with a line-broadening factor of 0.3 Hz and a zero filling (size = 64 K) procedure. ^1^H NMR spectra were manually phased. Chemical shifts were reported with respect to the residual CHCl_3_ signal set at 7.26 ppm. The baseline was corrected using the Cubic Spline Baseline Correction routine in the Bruker Topspin software.

The intensity of several selected signals (hexanal and trans 2-hexenal, terpene 2 and terpene 1, *sn*-1,3 diglycerides, *sn*-1,2 diglycerides, squalene, unsaturated fatty chains, saturated fatty chains, 24-methylene cycloartanol, linolenic fatty chains, linoleic fatty chains, β-sitosterol), see Table S2, was measured using the semi-automatic peak picking routine of Bruker TOPSPIN software and normalized with respect to the resonance at 2.251 ppm, due to α-methylenic protons of all acyl chains, normalized to 1000.

### 3.4. Statistical Analysis

NMR data were submitted to the STATISTICA software package for Windows. A statistical procedure based on the following points was performed:

- Analysis of variance (ANOVA). In this analysis the variables with the highest index of variability were selected according to their *p* level and *F* values. The *p* level represents a decreasing index of the reliability of a result and gives the probability of error involved in accepting a result as valid. A *p* level of 0.05 (5% probability of error) is usually treated as a borderline acceptable error level. The *F* value is the ratio between groups’ variability to within-group variability: The larger this ratio, the larger the discrimination power of the corresponding variable.
- Principal component analysis (PCA). PCA was performed on the variables with the highest index of variability: The percentage of variance for each specific principal component was reported. PCA results are shown reporting the scores of the principal components and also as a plot of the variable loadings.

## 4. Conclusions

The investigation carried out in the present work offers a reliable protocol that can play a significant role in the characterization and assessment quality of olive oils. This approach helped to describe olive oils of the “*Colline Pontine*” PDO area, which turned out to be characterized by specific sensory and chemical markers.

Therefore, the present findings suggest a positive contribution of this multidisciplinary analytical protocol for promoting the diffusion of high quality olive growing in the “*Colline Pontine*” PDO area. This methodology can give an important contribution in the PDO characterization and confirmation. This certification improves the value of the Mediterranean products and provides assurance that a product is of a high quality and has a defined origin.

## Figures and Tables

**Table 1:** Sensory profiles of olive oils. The defective samples are reported in bold type.

Code	Score	Fruity	Bitter	Pungent	Tomato	Fusty/Muddy Sediment	Musty/Humid/ Earthy	Winey/Vinegary/Acid/Sour	Metallic	Rancid	Defect
1	7.6	4.0	3.5	4.0	3.5	0.0	0.0	0.0	0.0	0.0	0.0
2	7.9	6.0	5.0	5.0	4.0	0.0	0.0	0.0	0.0	0.0	0.0
3	7.7	5.0	4.5	4.0	4.0	0.0	0.0	0.0	0.0	0.0	0.0
4	7.0	4.0	3.8	4.0	2.5	0.0	0.0	0.0	0.0	0.0	0.0
5	8.1	6.0	4.5	5.0	4.5	0.0	0.0	0.0	0.0	0.0	0.0
6	7.0	4.0	3.0	3.0	0.0	0.0	0.0	0.0	0.0	0.0	0.0
7	7.0	4.0	3.0	3.2	1.0	0.0	0.0	0.0	0.0	0.0	0.0
**8**	**6.0**	**3.0**	**3.0**	**2.5**	**0.0**	**2.0**	**0.0**	**0.0**	**0.0**	**0.0**	**2.0**
**9**	**6.9**	**4.0**	**4.0**	**3.0**	**0.0**	**0.0**	**1.0**	**0.0**	**0.0**	**0.0**	**1.0**
10	7.6	4.5	3.5	3.5	3.0	0.0	0.0	0.0	0.0	0.0	0.0
11	7.8	5.5	5.0	4.8	4.5	0.0	0.0	0.0	0.0	0.0	0.0
**12**	**6.0**	**3.0**	**2.0**	**3.0**	**0.0**	**1.5**	**0.0**	**0.0**	**0.0**	**0.0**	**1.5**
13	7.7	5.0	4.0	4.5	4.0	0.0	0.0	0.0	0.0	0.0	0.0
14	8.1	6.5	5.0	4.7	4.9	0.0	0.0	0.0	0.0	0.0	0.0
15	8.4	6.5	5.5	5.5	4.0	0.0	0.0	0.0	0.0	0.0	0.0
16	7.9	3.8	3.5	3.7	1.1	0.0	0.0	0.0	0.0	0.0	0.0
17	7.8	5.1	5.0	5.0	2.5	0.0	0.0	0.0	0.0	0.0	0.0
18	7.3	3.7	2.9	2.9	1.2	0.0	0.0	0.0	0.0	0.0	0.0
19	8.3	5.9	5.1	5.3	3.4	0.0	0.0	0.0	0.0	0.0	0.0
20	8.1	5.9	5.0	5.0	3.4	0.0	0.0	0.0	0.0	0.0	0.0
21	7.1	3.8	3.6	3.3	2.0	0.0	0.0	0.0	0.0	0.0	0.0
22	7.8	5.2	3.9	4.2	2.9	0.0	0.0	0.0	0.0	0.0	0.0
23	7.7	4.8	4.0	4.0	3.0	0.0	0.0	0.0	0.0	0.0	0.0
24	8.4	6.2	5.6	5.4	4.0	0.0	0.0	0.0	0.0	0.0	0.0
**25**	**6.7**	**2.4**	**1.8**	**2.3**	**0.0**	**1.0**	**0.0**	**0.0**	**0.0**	**0.0**	**1.0**
26	7.5	4.1	3.5	4.1	3.1	0.0	0.0	0.0	0.0	0.0	0.0
27	8.0	5.1	4.5	5.0	3.0	0.0	0.0	0.0	0.0	0.0	0.0
28	7.2	4.0	3.2	3.3	1.0	0.0	0.0	0.0	0.0	0.0	0.0
29	7.4	4.0	3.2	3.2	2.4	0.0	0.0	0.0	0.0	0.0	0.0
30	7.4	4.5	4.0	4.1	1.3	0.0	0.0	0.0	0.0	0.0	0.0
31	7.8	4.0	3.5	4.3	2.5	0.0	0.0	0.0	0.0	0.0	0.0
32	7.5	4.5	3.1	3.8	2.7	0.0	0.0	0.0	0.0	0.0	0.0
**33**	**6.7**	**3.5**	**3.0**	**3.3**	**0.0**	**0.0**	**0.0**	**1.0**	**0.0**	**0.0**	**1.0**
34	7.8	3.8	3.7	3.8	3.2	0.0	0.0	0.0	0.0	0.0	0.0
35	7.7	5.3	4.2	4.5	3.1	0.0	0.0	0.0	0.0	0.0	0.0
36	7.0	5.0	3.8	4.0	2.3	0.0	0.0	0.0	0.0	0.0	0.0
**37**	**6.0**	**3.0**	**2.0**	**1.6**	**0.2**	**1.2**	**0.0**	**0.0**	**0.0**	**2.0**	**2.0**
38	7.6	5.4	3.8	4.3	2.5	0.0	0.0	0.0	0.0	0.0	0.0
39	7.6	4.5	4.8	5.0	2.5	0.0	0.0	0.0	0.0	0.0	0.0
40	7.1	4.0	3.3	3.0	2.0	0.0	0.0	0.0	0.0	0.0	0.0

**Table 2:** δ^2^H, δ^18^O and δ^13^C of olive oils.

	Mean	Sd	Min	Max
δ^2^H ‰ vs. V-SMOW	−142	4	−152	−135
δ^18^O ‰ vs. V-SMOW	24.0	1.1	22.0	26.3
δ^13^C ‰ vs. V-PDB	−30.2	1.0	−31.9	−28.1

**Table 3:** The *sn*-1,2/*sn*-1,3 diglycerides ratio obtained from ^1^H-NMR normalized signal intensities.

Code	Ratio *sn*1,2/*sn*1,3	Code	Ratio *sn*1,2/*sn*1,3	Code	Ratio *sn*1,2/*sn*1,3	Code	Ratio *sn*1,2/*sn*1,3
1	41.30	21	46.00	41	13.83	61	6.74
2	56.75	22	12.86	42	17.49	62	4.53
3	44.33	23	33.20	43	14.74	-	-
4	19.92	24	39.58	44	13.82	-	-
5	35.30	25	31.22	45	9.20	-	-
6	57.89	26	31.25	46	12.63	-	-
7	49.60	27	47.67	47	11.84	-	-
8	5.73	28	15.57	48	16.06	-	-
9	17.21	29	25.12	49	6.80	-	-
10	65.29	30	38.22	50	29.74	-	-
11	43.67	31	57.00	51	20.38	-	-
12	17.88	32	41.69	52	20.73	-	-
13	33.53	33	24.14	53	11.58	-	-
14	40.09	34	51.17	54	5.60	-	-
15	37.33	35	35.64	55	11.75	-	-
16	27.32	36	27.00	56	8.26	-	-
17	47.40	37	70.50	57	9.48	-	-
18	37.92	38	18.20	58	11.43	-	-
19	65.33	39	57.91	59	5.74	-	-
20	46.70	40	30.46	60	7.59	-	-

## Supplementary Materials

The following are available online at http://www.mdpi.com/2218-1989/8/3/43/s1, Table S1: Characteristics of olive oil samples, Table S2: Chemical shifts and assignment of the thirteen NMR variables selected for ANOVA analysis.
