# Supplementary material for: A Multi-Methodological Protocol to Characterize PDO Olive Oils
Source: Metabolites. 2018 Jul 28;8(3):43. doi: 10.3390/metabo8030043 (PMC6160970; doi:10.3390/metabo8030043)
Supplement: Supplementary file 1 [file metabolites-08-00043-s001.pdf]

Table S1. Characteristics of olive oil samples.

Code	Geographical origin	Cultivar	Harvesting year
1	Priverno	Itrana	2011-2012
2	Sonnino	Itrana	2011-2012
3	Sonnino	Itrana	2011-2012
4	Terracina	Itrana	2011-2012
5	Lenola	Itrana	2011-2012
6	Gaeta	Itrana	2011-2012
7	SS Cosma e Damiano	Itrana	2011-2012
8	Cisterna di Latina	Itrana	2011-2012
9	Cisterna di Latina	Carolea	2011-2012
10	Rocca Massima	Itrana	2011-2012
11	Sonnino	Itrana	2011-2012
12	Maenza	Itrana	2011-2012
13	Maenza	Itrana	2011-2012
14	Sermoneta	Itrana	2011-2012
15	Priverno	Itrana	2012-2013
16	Sonnino	Itrana	2012-2013
17	Sonnino	Itrana	2012-2013
18	Terracina	Itrana	2012-2013
19	Lenola	Itrana	2012-2013
20	Gaeta	Itrana	2012-2013
21	SS Cosma e Damiano	Itrana	2012-2013
22	Cisterna di Latina	Itrana	2012-2013
23	Rocca Massima	Itrana	2012-2013
24	Sonnino	Itrana	2012-2013
25	Maenza	Itrana	2012-2013
26	Maenza	Itrana	2012-2013
27	Sermoneta	Itrana	2012-2013
28	Cisterna di Latina	Carolea	2012-2013
29	Spigno S.	Itrana	2012-2013
30	Spigno S.	Itrana	2012-2013
31	Cisterna di Latina	Itrana	2012-2013
32	Terracina	Itrana	2012-2013
33	Fondi	Itrana	2012-2013

34	Aprilia	Itrana	2012-2013
35	Itri	Itrana	2012-2013
36	Sonnino	Itrana	2012-2013
37	Terracina	Itrana	2012-2013
38	Itri	Itrana	2012-2013
39	Aprilia	Itrana, Frantoio	2012-2013
40	Terracina	Itrana	2012-2013
41	Priverno	Itrana	2013-2014
42	Sonnino	Itrana	2013-2014
43	Sonnino	Itrana	2013-2014
44	Terracina	Itrana	2013-2014
45	Lenola	Itrana	2013-2014
46	Gaeta	Itrana	2013-2014
47	SS Cosma e Damiano	Itrana	2013-2014
48	Rocca Massima	Itrana	2013-2014
49	Sonnino	Itrana	2013-2014
50	Maenza	Itrana	2013-2014
51	Minturno	Itrana	2013-2014
52	Sermoneta	Itrana	2013-2014
53	Castelforte	Itrana	2013-2014
54	Spigno S.	Itrana	2013-2014
55	Cisterna	Itrana	2013-2014
56	Terracina	Itrana	2013-2014
57	Lenola	Itrana	2013-2014
58	Aprilia	Itrana	2013-2014
59	Itri	Itrana	2013-2014
60	Sonnino	Itrana	2013-2014
61	Itri	Itrana, Frantoio	2013-2014
62	Formia	Itrana	2013-2014

Table S2. Chemical shifts and assignment of the thirteen NMR variables selected for ANOVA analysis.

	**Assignment**	**NMR Resonances (ppm)**
1	Hexanal	9.704
2	Trans 2-hexenal	9.454
3	Terpene 2	4.885
4	Terpene 1	4.541
5	Methynic protons in β-glycerol moiety of sn-1,3 diglycerides	3.988
6	Methylenic protons in α-glycerol moiety of sn-1,2 diglycerides	3.636
R	Reference peak due to methylenic protons bound to C2 normalized to 1000	2.251
7	Squalene	1.620
8	Methylenic protons of all unsaturated fatty chains	1.244
9	Methylenic protons of palmitic and stearic fatty chains	1.197
10	24-methylene cycloartanol	0.978
11	Methyl of linolenic fatty chains	0.910
12	Methyl of linoleic fatty chains	0.843
13	Methyl-18 of β-sitosterol	0.623
